# The High Flex Total Knee Arthroplasty—Higher Incidence of Aseptic Loosening and No Benefit in Comparison to Conventional Total Knee Arthroplasty: Minimum 16-Years Follow-Up Results

**DOI:** 10.1007/s43465-020-00276-3

**Published:** 2020-10-06

**Authors:** Florian Radetzki, Alexander Zeh, Karl-Stefan Delank, David Wohlrab

**Affiliations:** 1 Department of Orthopedic and Trauma Surgery, Dessau Medical Center, Brandenburg Medical School Theodor Fontane, Dessau, Auenweg 38, 06847 Dessau-Roßlau, Germany; 2 Medical Faculty, Martin Luther University Halle-Wittenberg, Magdeburger Straße 8, 06112 Halle (Saale), Germany; 3 Department of Orthopaedics, Trauma and Reconstructive Surgery, Martin Luther University Halle-Wittenberg, Ernst-Grube-Straße 40, 06120 Halle (Saale), Germany

**Keywords:** Knee arthroplasty, High-flex knee, Aseptic loosening, Flexion, Long-term results

## Abstract

**Purpose:**

This prospective randomized study compares the clinical and radiographic long-term results at least 16 years after total knee arthroplasty (TKA) between a mobile-bearing high flex and a fixed-bearing posterior-stabilized knee.

**Methods:**

In 2000, we included 60 patients who underwent a TKA. Patients were divided into two groups. At the time of the follow-up after a minimum of 16 years (16.5 years ± 0.6), 16 patients had died for causes unrelated to the operation, 15 were lost to the follow-up. Five patients of the high flex group had a revision. The final evaluation included the hospital for special surgery score (HSS) and radiographs. Using the X-rays and the Knee Society Roentgenographic Evaluation and Scoring System, radiolucent lines and the maximal knee flexion were determined.

**Results:**

No significant differences between the two groups were found. The mean HSS score of LPS group patients was 87.9 (± 10.6) points and that of the high flex group was 93.1 (± 7.4) points. Five patients of the high flex group had undergone a reoperation. One knee was revised for painful mid-flexion instability and the others for symptomatic aseptic loosening of the components.

**Conclusion:**

The long-term follow-up does not show any clear benefits and even a higher incidence of implant loosening using the mobile-bearing high-flex knee.

## Introduction

The high flex total knee arthroplasty (TKA) was developed for patients requiring a deeper flexion up to 150° for reasons of profession, culture and religion. Therefore, the high flex knee (NexGen LPS Flex mobile, Zimmer Inc., Warsaw, USA) was specially designed to prevent increasing wear and to maintain knee stability in comparison to the regular posterior stabilised (PS) knee (NexGen LPS). Therefore, it is a mobile-bearing system that allows an internal and external rotation of 25° each. An additional 2 mm bone cut from the posterior femoral condyles leads to a greater curvature of the posterior condyles. Furthermore, the tibial insert has an anterior cut to avoid patellar tendon impingement during deep flexion. The special design is expected to reduce contact stresses at the different interfaces and decreased polyethylene wear rate. Rotational mobility of the bearing surface might also optimize the tibio-femoral alignment, which could lead to a higher stability and increased anterior knee pain [1]. Wohlrab et al*.* and Radetzki et al. previously reported the short-, mid- and long-term results (3-month, 3-year, 5-year and 10-year follow-up) of a randomised clinical and radiological study comparing the results after TKA using the NexGen Flex mobile knee versus the NexGen LPS knee [2, 3]. 3- and 5-year postoperatively, there were no significant differences between both groups for the hospital for special surgery score (HSS) either, nor for the radiological results. A clear advantage of the mobile-bearing knee prostheses could not be reflected in the clinical results up to 5 years after surgery. Other studies had even shown a higher complication rate postoperatively using mobile-bearing systems. Ridgeway et al. observed early coronal plane instability and knee pain in 25 cases within 2 years postoperatively [4]*.* Further, subluxation, dislocation, increasing wear rate and stress shielding were observed [4]*.* The long-term results after 10 years have shown no clear benefits and even higher incidence of implant loosening of the high flex knee system [2]*.*

The aim of this study was to compare clinical and radiographic long-term results at least 16 years after of TKA using a high flex knee (NexGen Flex mobile) versus a regular PS knee (NexGen LPS). In addition, the study should also evaluate if the high flex knee system will cause additional implant loosening over time.

## Materials and Methods

In 2000, we included 60 patients who underwent a TKA in a prospective randomized study. Patients were divided into two groups by following a randomization list, preoperatively. In 30 patients, the NexGen LPS implant (Zimmer Inc., Warsaw, USA) was implanted. The remaining 30 patients received the high flex knee NexGen LPS Flex mobile (Zimmer Inc.). All patients in this study were diagnosed with unilateral degenerative arthritis, had a body mass index (BMI) less than 30, no previous joint infection, a varus deformity of less than 10° or a valgus deformity less than 5°. Preoperatively, we recorded patients’ data (age, gender, height, body weight) as well as the hospital for special surgery score (HSS) [5]. All surgeries were done by two well-trained surgeons. The surgeons were equally familiar with both knee system and their learning curves were completed. The operative procedures were similar in both the groups. In all procedures, a mid-vastus approach was used. The patella was resurfaced and all implants were cemented in all cases. Tourniquet was used in each patient. Drains were removed 48 h after surgery. The rehabilitation program during admission was the same for both the groups and the patients were discharged 5–7 days after surgery.

At the time of the follow-up after a minimum of 16 years (16.5 years ± 0.6), 16 patients (7 in the LPS group, 9 in the high flex group) had died for causes unrelated to the operation. Their preoperative average age was already 72.1 years and, therefore, well over 85 years at the time of the follow-up. Fifteen (8 in the LPS group, 7 in the high flex group) were lost to the follow-up. Five patients of the high flex group had undergone a reoperation of their knee. This left a total of 15 LPS knees and 9 high flex knees for final evaluation including the HSS score and radiographs (AP view in extension and lateral view in maximal flexion). Using the X-rays and the Knee Society Roentgenographic Evaluation and Scoring System [6], radiolucent lines and the maximal knee flexion were determined. Statistical analysis was performed with SPSS 22 software (SPSS Inc., Chicago, USA). Descriptive statistics presented include the mean and standard deviation (SD). A confidence interval of 95% was assumed (significance level *p* < 0.05). The data were analysed with Student’s *t* test.

## Results

Preoperatively, there were no relevant differences between the two groups. The mean HSS score of LPS group patients was 48.6 (± 8.5) points and that of the high flex group was 54.4 (± 6.3) points. In the subgroups of the HSS score, there were no differences except for the category pain that was less in the high flex group. There were also no relevant differences in the maximal knee flexion between the two groups preoperatively (Table 1).

**Table 1:** Demographic data, HSS and radiographic evaluation preoperative and 16 years after surgery

	Preoperatively				16 years			
	LPS		High flex		LPS		High flex	
	Mean	SD	Mean	SD	Mean	SD	Mean	SD
*N* (joints)	30	–	30	–	15	–	9	–
Age	65.5	9.1	66.5	5.3	80.7	7.9	78.7	10.9
Male	12	–	14	–	7	–	2	–
Female	18	–	16	–	8	–	7	–
BMI	24.4	6.6	24.1	5.9	30.1	5.2	29.1	3.7
HSS score								
Pain	3.0	4.8	8.0	3.1	27.0	3.7	28.9	3.3
Function	12.8	3.2	14.1	2.2	21.2	2.4	21.7	0.7
Range of motion	13.4	2.0	13.7	1.6	14.5	1.7	14.8	1.2
Muscle strength	9.2	1.4	9.9	0.5	9.9	0.5	10.0	0.0
Flexion deformity	6.3	2.9	5.0	2.6	7.0	4.1	8.3	3.5
Instability	8.2	1.2	7.4	1.1	9.3	1.4	9.6	0.9
Substraction	4.4	2.1	3.8	2.2	0.9	1.2	0.2	0.7
Total HSS	48.6	8.5	54.4	6.3	87.9	10.6	93.1	7.4
Radiological evaluation								
Maximal flexion	105.6	17.8	108.5	14.8	113.5	11.1	115.7	8.5

At the 16-year follow-up, there were no significant differences between the two groups regarding the clinical and radiographic results. The mean HSS score of LPS group patients was 87.9 (± 10.6) points and that of the high flex group was 93.1 (± 7.4) points (*p* = 0.21). Especially the theoretical advantages of high flex knee systems, namely the range of motion (high flex 14.8, LPS 14.5, *p* = 0.71), joint stability (high flex 9.6, LPS 9.3, *p* = 0.59) and function (high flex 21.7, LPS 21.2, *p* = 0.46) were comparable. In the radiological evaluation, the maximal flexion of the high flex group was 115.7° (± 8.5) and that of the LPS group 113.5 (± 11.1) (*p* = 0.52) (Table 1).

Using the Knee Society Roentgenographic Evaluation and Scoring System, there was one patient of the LPS group with radiolucent lines smaller than 10 mm in zones 1 and 4 of the tibia in the AP view without clinical symptoms and a HSS score of 91.

**Fig. 1:**
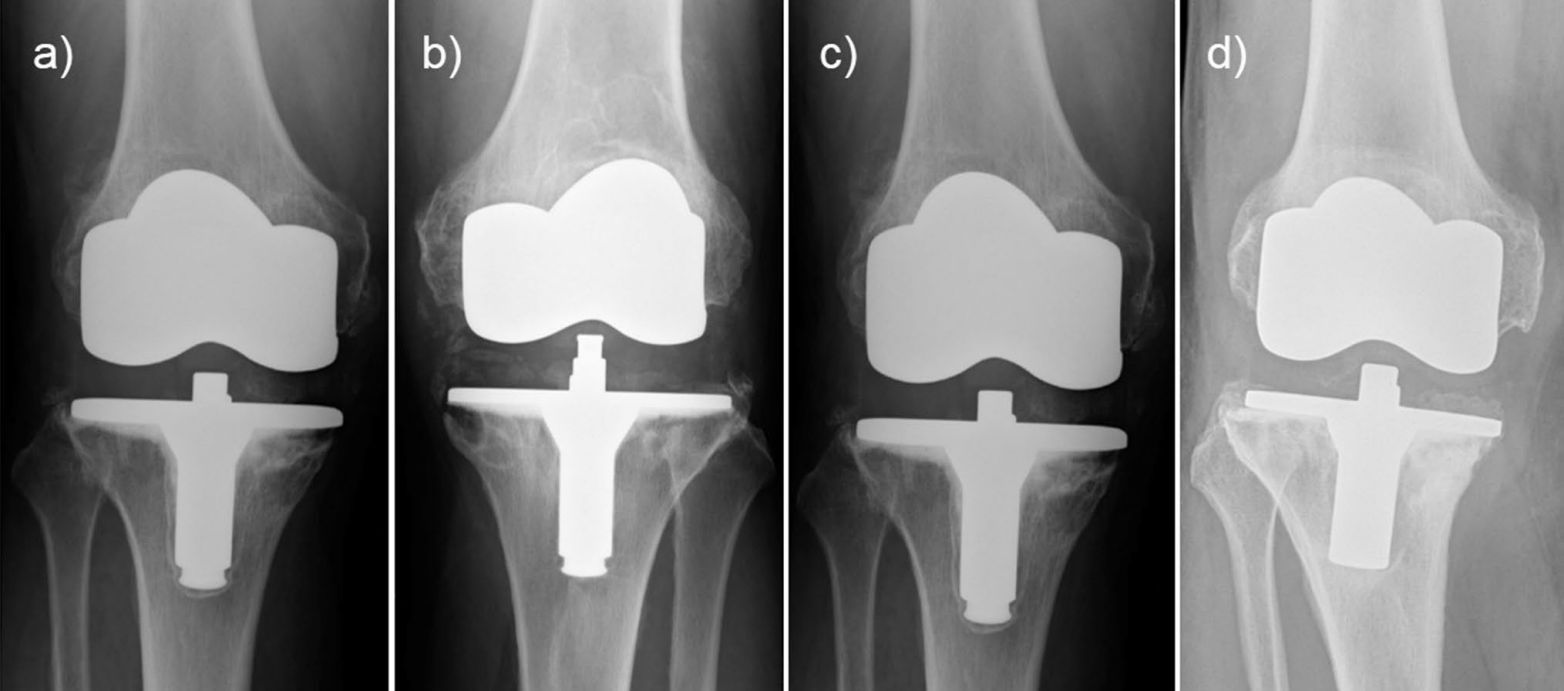
High-flex knees with aseptic loosening of the cemented tibial prosthesis after **a** 7 years (zone 1 and 5), **b** 8 years (zone 1), **c** 14 years (zone 1), and **d** 16 years (all zones with dislocation) using the Knee Society Roentgenographic Evaluation and Scoring System

Total revision surgeries were performed in 5 knees, all in the high flex group (16.7%). One patient of the high flex group had a painful mid-flexion instability that needed to be revised after 5 years. The mean revision rate due to aseptic loosening was 4 of 30 knees (13.3%) and all were men. Because of symptomatic aseptic loosening of the cemented tibial prosthesis, four high flex knees were revised after 7, 8, 14 and 16 years. In the AP view, tibial radiolucent lines were found just in zone 1 for two knees, zone 1 and 5 in a second knee and another showed a progressive lucency in all zones with dislocation, as described in the Knee Society Roentgenographic Evaluation and Scoring System (Fig. 1). There was no noticed revision in the LPS group.

## Discussion

**Fig. 2:**
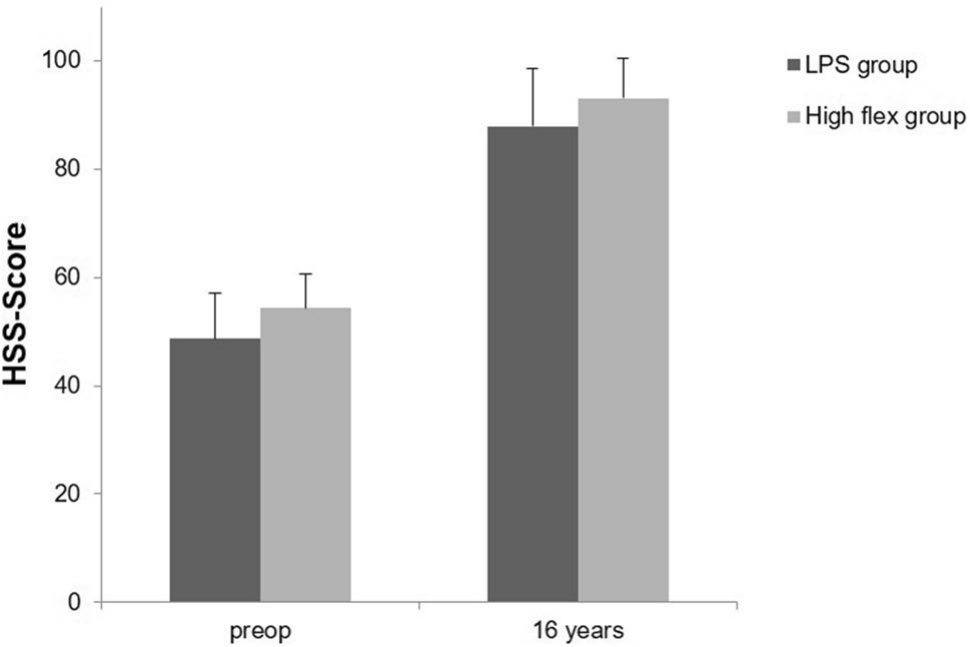
Total HSS of the NexGen LPS (LPS group) versus NexGen LPS Flex mobile (high flex group), pre- and postoperatively

**Fig. 3:**
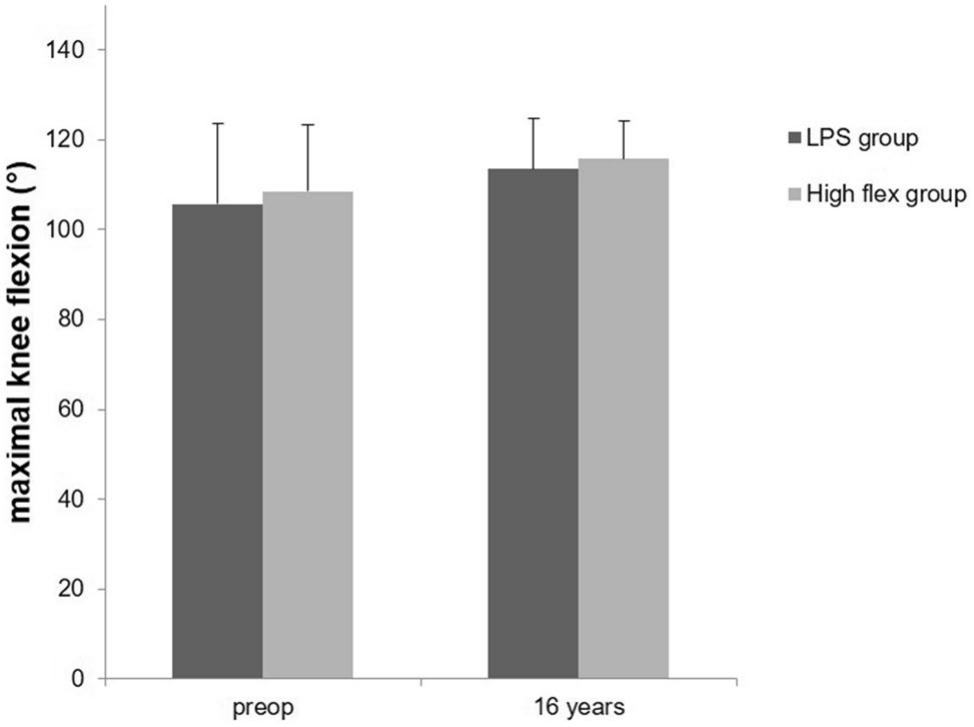
Maximal knee flexion of the NexGen LPS (LPS group) versus NexGen LPS Flex mobile (high flex group) measured using X-rays of the knee in lateral view

The high flex total knee arthroplasty with a mobile-bearing system was developed to increase the ROM and to optimize the tibio-femoral alignment (Figs. 2, 3). This self-alignment should theoretically decrease the wear rate with a positive effect in preventing early implant loosening. Several studies had shown that these advantages are not reproducible in clinical practice in considering short- and mid-term results [1, 3, 4, 7]*.* In the presented study, we compared the clinical and radiographic long-term results of the high flex knee (NexGen Flex mobile, Zimmer Inc.) and regular PS knee (NexGen LPS, Zimmer Inc.) after a minimum of 16 years. Wohlrab et al. already reported the short- and mid-term results (3-month, 3-year and 5-year follow-up) of both study groups [3]. Three months postoperatively, there were better results in scores for pain, ROM (122.5° vs. 107.33°), as well as in the overall HSS (87.21 vs. 82.68 points) in the high flex group. 3, 5 and 10 years postoperatively, there were no significant differences between both groups for the hospital for special surgery score (HSS) either, nor for the radiological results [2, 3].

After 16 years, we found no differences between patients with these implant designs with respect to pain, function, range of motion, muscle strength, flexion deformity and instability, as well. The theoretical benefit of high flex knee systems, namely the ROM (113.5 vs. 108.3), joint stability and function were still similar. Choi et al. did also find no significant differences between the two groups regarding postoperative total arc of knee motion, Knee Society score and function score [8]. In the long-term follow-up study after a minimum of 20 years, Kim determined comparable outcomes and survivorship for the high-flexion knee in comparison to a standard knee as well [9]. On the other hand, several studies have reported an improved ROM comparing standard designs with high flex designs within the first two years after surgery [10–13]. Anyway, the majority of prospective randomized trials showed no significant difference comparing high flex knee system to conventional knee system [3, 14–16]. It is assumed that the preselection of patients with at least 90° of flexion and not excessively high BMIs is jointly responsible for the clinical results with respect to function and flexion [16].

Instability and subluxation are typical complications especially with mobile-bearing systems [4, 17]. We just revised one patient of the high flex group for painful mid-flexion instability after 5 years.

It is postulated that mobile-bearing systems could reduce contact stresses at the different interfaces, resulting in a decrease of the wear rate with a positive effect in preventing early implant loosening [18–20]. The results of our follow-up after a minimum of 16 years cannot confirm this advantage. Choi et al. also observed a significantly higher revision rate due to aseptic loosening for the high flex knee (4.9%) in comparison to the conventional knee (0.6%) in a large population in the mid-term follow-up [8]. Han et al. reported a 38% aseptic loosening rate in 72 high-flexion knees at a mean follow-up of 32 months as well [21]. Critical stress was noticed at the femoral fixation site at high-flexion angle and lead to early loosening of the femoral component in high-flexion knees [22]. We were not able to confirm this association having more tibial than femoral loosening in our series. Kim et al. had two knees (0.2%) in the high-flexion knee group undergoing a revision of both femoral and tibial components after a mean of 13.2 years. An increased incidence of femoral component loosening in the high-flexion knee group could not be found in that study [23].

The major weakness of this study is the high number of lost patients in each group. All in terms, 15 (8 in the LPS group, 7 in the high flex group) knees (25%) were lost to the final follow-up although an extensive effort in contacting all patients was undertaken. However, it is a quite obvious limitation and inherent difficulty in dealing with long-term studies [4]. In addition, 16 patients died until the 16-year examination. An already old age of these patients at the beginning of the study limits the long-term control. As a consequence, the amount of included patients will have to be increased right from the beginning of long-term follow-ups in the future.

In summary, this prospective, randomized controlled trial demonstrated no benefit to the high flex knee system versus a regular PS knee up to 16 years after surgery. Five patients of the high flex group had to be revised for reasons of instability and aseptic implant loosening. There were no revisions in the LPS group.

The long-term follow-up does not show any clear benefits and even a higher incidence of implant loosening using the high flex knee (NexGen Flex mobile). It must be seriously revaluated and used carefully considering the higher revision rate. Further studies are required to prove long-term adverse effects of high flex knee systems.
