# Correction to: The High Flex Total Knee Arthroplasty—Higher Incidence of Aseptic Loosening and No Benefit in Comparison to Conventional Total Knee Arthroplasty: Minimum 16-Years Follow-Up Results

**DOI:** 10.1007/s43465-021-00449-8

**Published:** 2021-08-16

**Authors:** Florian Radetzki, Alexander Zeh, Karl-Stefan Delank, David Wohlrab

**Affiliations:** 1 Department of Orthopedic and Trauma Surgery, Dessau Municipal Hospital, Auenweg 38, 06847 Dessau-Roßlau, Germany; 2 Medical Faculty, Martin Luther University Halle-Wittenberg, Magdeburger Straße 8, 06112 Halle (Saale), Germany; 3 Department of Orthopaedics, Trauma and Reconstructive Surgery, Martin Luther University Halle-Wittenberg, Ernst-Grube-Straße 40, 06120 Halle (Saale), Germany

## Correction to: Indian Journal of Orthopaedics (2021) 55(Suppl 1):S76–S80 10.1007/s43465-020-00276-3

The article “The High Flex Total Knee Arthroplasty—Higher Incidence of Aseptic Loosening and No Benefit in Comparison to Conventional Total Knee Arthroplasty: Minimum 16-Years Follow-Up Results”, written by Radetzki, F., Zeh, A., Delank, K.-S. and Wohlrab, D., was originally published Online First without Open Access. After publication in volume 55, supplement 1, pages S76–S80, the author decided to opt for Open Choice and to make the article an Open Access publication. Therefore, the copyright of the article has been changed to © The Author(s) 2020 and the article is forthwith distributed under the terms of the Creative Commons Attribution 4.0 International License, which permits use, sharing, adaptation, distribution and reproduction in any medium or format, as long as you give appropriate credit to the original author(s) and the source, provide a link to the Creative Commons licence, and indicate if changes were made. The images or other third party material in this article are included in the article’s Creative Commons licence, unless indicated otherwise in a credit line to the material. If material is not included in the article's Creative Commons licence and your intended use is not permitted by statutory regulation or exceeds the permitted use, you will need to obtain permission directly from the copyright holder. To view a copy of this licence, visit http://creativecommons.org/licenses/by/4.0/.

The original article has been corrected.

