# Correction to: The High Flex Total Knee Arthroplasty—Higher Incidence of Aseptic Loosening and No Benefit in Comparison to Conventional Total Knee Arthroplasty: Minimum 16-Years Follow-Up Results

**DOI:** 10.1007/s43465-022-00615-6

**Published:** 2022-03-11

**Authors:** Florian Radetzki, Alexander Zeh, Karl-Stefan Delank, David Wohlrab

**Affiliations:** 1 Department of Orthopedic and Trauma Surgery, Dessau Medical Center, Brandenburg Medical School Theodor Fontane, Dessau, Auenweg 38, 06847 Dessau-Roßlau, Germany; 2 Medical Faculty, Martin Luther University Halle-Wittenberg, Magdeburger Straße 8, 06112 Halle (Saale), Germany; 3 Department of Orthopaedics, Trauma and Reconstructive Surgery, Martin Luther University Halle-Wittenberg, Ernst-Grube-Straße 40, 06120 Halle (Saale), Germany

## Correction to: Indian Journal of Orthopaedics (2021) 55 (Suppl 1):S76–S80 10.1007/s43465-020-00276-3

In this article, the details of affiliation 1 were incorrectly given as “Department of Orthopedic and Trauma Surgery, Dessau Municipal Hospital, Auenweg 38, 06847 Dessau-Roßlau, Germany” but should have been “Department of Orthopedic and Trauma Surgery, Dessau Medical Center, Brandenburg Medical School Theodor Fontane, Dessau, Auenweg 38, 06847 Dessau-Roßlau, Germany”.

The original article has been corrected.

